# {1,3-Bis[(diphenyl­phosphanyl-κ*P*)­oxy]propane}dicarbonyl­iron(0)

**DOI:** 10.1107/S1600536810013759

**Published:** 2010-04-21

**Authors:** Guoqiang Xu, Xiaoyan Li

**Affiliations:** a School of Chemistry and Chemical Engineering, Shandong University, Shanda Nanlu 27, Jinan 250100, People’s Republic of China

## Abstract

The structure of the title compound, [Fe(C_27_H_26_O_2_P_2_)(CO)_2_], exhibits a distorted tetra­hedral coordination [bond angle range = 96.31 (12)–119.37 (4)°], comprising two P-atom donors from the chelating 1,3-bis­[(diphenyl­phosphan­yl)­oxy]propane ligand [Fe—P = 2.1414 (10) and 2.1462 (10) Å] and two carbonyl ligands [Fe—C = 1.763 (4) and 1.765 (3) Å].

## Related literature

For a related carbonyl­ation reaction, see: Klein *et al.* (2003 ▶). For general background to metal complexes with the 1,3-bis­[(diphenyl­phosphino)­oxy]propane ligand, see: Pandarus *et al.* (2008 ▶); Xu *et al.* (2009 ▶).
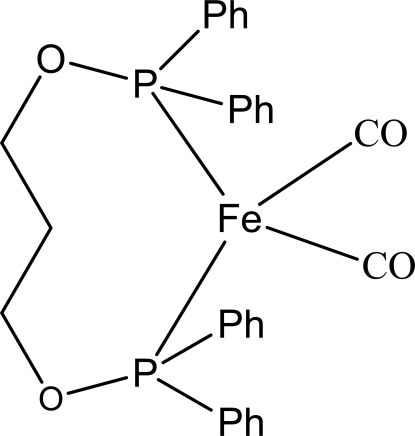


## Experimental

### 

#### Crystal data

[Fe(C_27_H_26_O_2_P_2_)(CO)_2_]
*M* *_r_* = 556.29
Monoclinic, 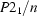

*a* = 12.589 (3) Å
*b* = 15.191 (3) Å
*c* = 14.384 (3) Å
β = 106.14 (3)°
*V* = 2642.4 (11) Å^3^
*Z* = 4
Mo *K*α radiation
μ = 0.73 mm^−1^
*T* = 293 K
0.27 × 0.20 × 0.15 mm

#### Data collection

Bruker SMART CCD area-detector diffractometer
Absorption correction: multi-scan (*SADABS*; Sheldrick, 1996 ▶) *T*_min_ = 0.828, *T*_max_ = 0.899
17176 measured reflections
5590 independent reflections
3864 reflections with *I* > 2σ(*I*)
*R*_int_ = 0.080

#### Refinement

*R*[*F* ^2^ > 2σ(*F* ^2^)] = 0.049
*wR*(*F* ^2^) = 0.094
*S* = 0.99
5590 reflections
325 parameters
H-atom parameters constrained
Δρ_max_ = 0.55 e Å^−3^
Δρ_min_ = −0.30 e Å^−3^

### 

Data collection: *SMART* (Bruker, 1997 ▶); cell refinement: *SAINT* (Bruker, 1997 ▶); data reduction: *SAINT*; program(s) used to solve structure: *SHELXS97* (Sheldrick, 2008 ▶); program(s) used to refine structure: *SHELXL97* (Sheldrick, 2008 ▶); molecular graphics: *SHELXTL* (Sheldrick, 2008 ▶); software used to prepare material for publication: *SHELXTL*.

## Supplementary Material

Crystal structure: contains datablocks I, global. DOI: 10.1107/S1600536810013759/zs2034sup1.cif

Structure factors: contains datablocks I. DOI: 10.1107/S1600536810013759/zs2034Isup2.hkl

Additional supplementary materials:  crystallographic information; 3D view; checkCIF report

## Figures and Tables

**Fig. 1.**
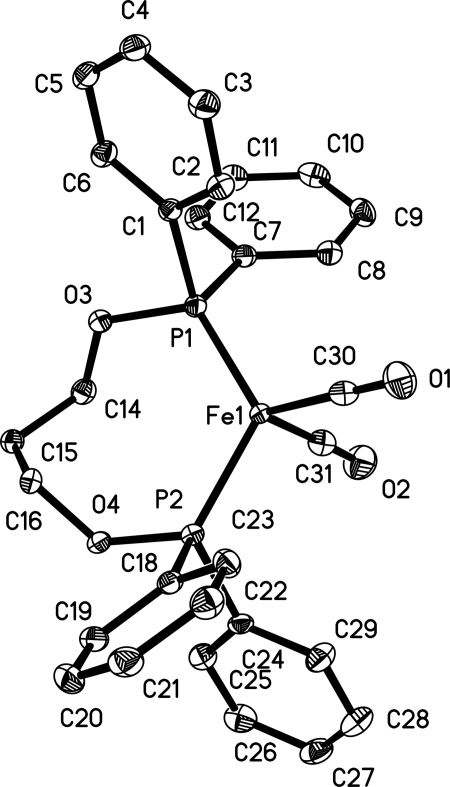
Atom numbering scheme for (I) with non-H atoms shown as 30% probability displacement ellipsoids.

## supplementary crystallographic information

### Comment

In recent years, the three-coordinated phosphine-based pincer (PCP) ligands with an aliphatic backbone have attracted much attention (Pandarus *et al.*, 2008). Sometimes the ligand backbone has been found to be too flexibile to be metallated. We have reported that the central sp^3^ C—H bond of (Ph_2_POCH_2_)_2_CH_2_ could be activated by Fe(PMe_3_)_4_Me_2_ to afford metallated PCP pincer compounds at room temperature (Xu *et al.*, 2009). When a solution of (Ph_2_POCH_2_)_2_CH_2_ was mixed with Fe(PMe_3_)_4_, and then placed under an atmosphere of CO at 273 K, the title compound [1,3-bis((diphenylphosphino)oxy)propane]bis(carbonyl)iron(0) (I) was formed. In this, the PMe_3_ ligands were replaced by CO. A similar carbonylation reaction was reported by Klein *et al.* (2003).

The molecular structure of the title compound is shown in Fig. 1. The Fe is coordinated by two phosphane P atoms from the chelating (Ph_2_POCH_2_)_2_CH_2_ ligand and two carbonyl C atoms, the complex exhibiting a distorted tetrahedral stereochemistry [Fe–P, 2.1414 (10), 2.1462 (10) Å; Fe–C, 1.763 (4), 1.765 (3) Å; bond angle range, 96.31 (12)–119.37 (4)°].

### Experimental

Standard vacuum techniques were used in manipulations of volatile and air sensitive material. The title compound was synthesized by combining a solution of (Ph_2_POCH_2_)_2_CH_2_ (406 mg, 0.90 mmol) in 20 ml of diethylether with Fe(PMe_3_)_4_ (324 mg, 0.90 mmol) in 20 ml of diethylether at 273 K, at which time the color changed from yellow to red. After stirring for 12 h, the solution was placed under an atmosphere of CO at 273 K for a further 12 h, resulting in a color change back to yellow, after which the solution was concentrated and filtered. Yellow crystals (310 mg, 62% yield) were obtained by recrystallization from a diethylether solution maintained at 253 K.

### Refinement

Hydrogen atoms were included in the refinement at calculated positions (C–H_aromatic_ = 0.93 Å; C–H_aliphatic_ = 0.97 Å) and treated as riding models, with *U*iso(H) = 1.2 (1.5 for alkyl groups) times *U*eq(C).

### Crystal data

**Table:**

[Fe(C_27_H_26_O_2_P_2_)(CO)_2_]	*F*(000) = 1152
*M**_r_* = 556.29	*D*_x_ = 1.398 Mg m^−^^3^
Monoclinic, *P*2_1_/*n*	Melting point: 385 K
Hall symbol: -P 2yn	Mo *K*α radiation, λ = 0.71073 Å
*a* = 12.589 (3) Å	Cell parameters from 17176 reflections
*b* = 15.191 (3) Å	θ = 1.9–26.8°
*c* = 14.384 (3) Å	µ = 0.73 mm^−^^1^
β = 106.14 (3)°	*T* = 293 K
*V* = 2642.4 (11) Å^3^	Block, yellow
*Z* = 4	0.27 × 0.20 × 0.15 mm

### Data collection

**Table:**

Bruker SMART CCD area-detector diffractometer	5590 independent reflections
Radiation source: fine-focus sealed tube	3864 reflections with *I* > 2σ(*I*)
graphite	*R*_int_ = 0.080
φ and ω scans	θ_max_ = 26.8°, θ_min_ = 1.9°
Absorption correction: multi-scan (*SADABS*; Sheldrick, 1996)	*h* = −15→14
*T*_min_ = 0.828, *T*_max_ = 0.899	*k* = −19→15
17176 measured reflections	*l* = −18→18

### Refinement

**Table:**

Refinement on *F*^2^	Primary atom site location: structure-invariant direct methods
Least-squares matrix: full	Secondary atom site location: difference Fourier map
*R*[*F*^2^ > 2σ(*F*^2^)] = 0.049	Hydrogen site location: inferred from neighbouring sites
*wR*(*F*^2^) = 0.094	H-atom parameters constrained
*S* = 0.99	*w* = 1/[σ^2^(*F*_o_^2^) + (0.035*P*)^2^] where *P* = (*F*_o_^2^ + 2*F*_c_^2^)/3
5590 reflections	(Δ/σ)_max_ = 0.001
325 parameters	Δρ_max_ = 0.55 e Å^−^^3^
0 restraints	Δρ_min_ = −0.30 e Å^−^^3^

### Special details

**Table:**

Geometry. All esds (except the esd in the dihedral angle between two l.s. planes) are estimated using the full covariance matrix. The cell esds are taken into account individually in the estimation of esds in distances, angles and torsion angles; correlations between esds in cell parameters are only used when they are defined by crystal symmetry. An approximate (isotropic) treatment of cell esds is used for estimating esds involving l.s. planes.
Refinement. Refinement of *F*^2^ against ALL reflections. The weighted *R*-factor wR and goodness of fit *S* are based on *F*^2^, conventional *R*-factors *R* are based on *F*, with *F* set to zero for negative *F*^2^. The threshold expression of *F*^2^ > σ(*F*^2^) is used only for calculating *R*-factors(gt) etc. and is not relevant to the choice of reflections for refinement. *R*-factors based on *F*^2^ are statistically about twice as large as those based on *F*, and *R*- factors based on ALL data will be even larger.

### Fractional atomic coordinates and isotropic or equivalent isotropic displacement parameters (Å^2^)

**Table:**

	*x*	*y*	*z*	*U*_iso_*/*U*_eq_	
Fe1	0.86496 (4)	0.14441 (3)	0.60132 (3)	0.01955 (11)	
P1	0.80915 (7)	0.11051 (5)	0.72478 (5)	0.02269 (17)	
P2	0.94395 (6)	0.26876 (5)	0.59809 (5)	0.02229 (17)	
C1	0.6609 (3)	0.1013 (2)	0.7101 (2)	0.0233 (6)	
C2	0.5887 (3)	0.0808 (2)	0.6205 (2)	0.0310 (7)	
H2	0.6157	0.0750	0.5669	0.037*	
C3	0.4769 (3)	0.0689 (3)	0.6105 (2)	0.0363 (8)	
H3	0.4296	0.0545	0.5503	0.044*	
C4	0.4353 (3)	0.0783 (2)	0.6893 (2)	0.0337 (8)	
H4	0.3603	0.0702	0.6825	0.040*	
C5	0.5061 (3)	0.0997 (2)	0.7781 (2)	0.0314 (7)	
H5	0.4782	0.1069	0.8311	0.038*	
C6	0.6177 (3)	0.1107 (2)	0.7895 (2)	0.0281 (7)	
H6	0.6645	0.1245	0.8501	0.034*	
C7	0.8558 (3)	0.0063 (2)	0.7873 (2)	0.0262 (7)	
C8	0.8773 (3)	−0.0665 (2)	0.7358 (2)	0.0310 (7)	
H8	0.8735	−0.0605	0.6706	0.037*	
C9	0.9041 (3)	−0.1472 (2)	0.7806 (2)	0.0361 (8)	
H9	0.9192	−0.1949	0.7458	0.043*	
C10	0.9086 (3)	−0.1566 (2)	0.8773 (2)	0.0356 (8)	
H10	0.9250	−0.2112	0.9071	0.043*	
C11	0.8887 (3)	−0.0852 (2)	0.9300 (2)	0.0342 (8)	
H11	0.8927	−0.0916	0.9952	0.041*	
C12	0.8629 (3)	−0.0040 (2)	0.8852 (2)	0.0290 (7)	
H12	0.8502	0.0440	0.9208	0.035*	
C14	0.9435 (3)	0.2174 (2)	0.8523 (2)	0.0280 (7)	
H14A	0.9913	0.1969	0.8143	0.034*	
H14B	0.9749	0.1985	0.9188	0.034*	
C15	0.9355 (3)	0.3167 (2)	0.8480 (2)	0.0276 (7)	
H15A	0.8859	0.3362	0.8847	0.033*	
H15B	1.0079	0.3416	0.8779	0.033*	
C16	0.8941 (3)	0.3508 (2)	0.7457 (2)	0.0267 (6)	
H16A	0.8238	0.3236	0.7135	0.032*	
H16B	0.8834	0.4140	0.7464	0.032*	
C18	0.8717 (2)	0.3468 (2)	0.5048 (2)	0.0246 (6)	
C19	0.8872 (3)	0.4367 (2)	0.5213 (2)	0.0295 (7)	
H19	0.9362	0.4564	0.5785	0.035*	
C20	0.8308 (3)	0.4973 (2)	0.4537 (2)	0.0347 (8)	
H20	0.8409	0.5572	0.4662	0.042*	
C21	0.7590 (3)	0.4685 (2)	0.3673 (2)	0.0350 (8)	
H21	0.7213	0.5090	0.3215	0.042*	
C22	0.7440 (3)	0.3796 (3)	0.3498 (2)	0.0352 (8)	
H22	0.6965	0.3602	0.2918	0.042*	
C23	0.7994 (3)	0.3186 (2)	0.4183 (2)	0.0294 (7)	
H23	0.7881	0.2587	0.4062	0.035*	
C24	1.0822 (3)	0.2664 (2)	0.5825 (2)	0.0241 (6)	
C25	1.1746 (3)	0.2932 (2)	0.6554 (2)	0.0309 (7)	
H25	1.1658	0.3147	0.7134	0.037*	
C26	1.2795 (3)	0.2883 (3)	0.6426 (3)	0.0376 (8)	
H26	1.3408	0.3058	0.6920	0.045*	
C27	1.2931 (3)	0.2572 (3)	0.5554 (3)	0.0401 (9)	
H27	1.3632	0.2545	0.5460	0.048*	
C28	1.2025 (3)	0.2307 (3)	0.4837 (2)	0.0412 (9)	
H28	1.2115	0.2097	0.4256	0.049*	
C29	1.0982 (3)	0.2345 (3)	0.4964 (2)	0.0365 (8)	
H29	1.0377	0.2156	0.4471	0.044*	
C30	0.7896 (3)	0.1023 (2)	0.4877 (2)	0.0328 (8)	
C31	0.9862 (3)	0.0802 (2)	0.6249 (2)	0.0351 (8)	
O1	0.7392 (2)	0.0776 (2)	0.41268 (18)	0.0520 (7)	
O2	1.0661 (2)	0.0395 (2)	0.6391 (2)	0.0513 (7)	
O3	0.83459 (18)	0.18051 (15)	0.81476 (13)	0.0262 (5)	
O4	0.97462 (18)	0.33011 (14)	0.69448 (14)	0.0261 (5)	

### Atomic displacement parameters (Å^2^)

**Table:**

	*U*^11^	*U*^22^	*U*^33^	*U*^12^	*U*^13^	*U*^23^
Fe1	0.0221 (2)	0.0190 (2)	0.02022 (18)	−0.0018 (2)	0.01025 (15)	−0.00077 (17)
P1	0.0277 (4)	0.0196 (4)	0.0234 (3)	0.0002 (3)	0.0115 (3)	0.0013 (3)
P2	0.0239 (4)	0.0228 (4)	0.0225 (3)	−0.0020 (3)	0.0104 (3)	−0.0002 (3)
C1	0.0275 (17)	0.0175 (15)	0.0284 (14)	0.0019 (13)	0.0136 (12)	0.0040 (12)
C2	0.0341 (19)	0.0316 (18)	0.0306 (15)	−0.0026 (15)	0.0145 (13)	−0.0005 (13)
C3	0.033 (2)	0.039 (2)	0.0369 (17)	−0.0055 (16)	0.0100 (14)	−0.0038 (15)
C4	0.0288 (19)	0.0318 (19)	0.0442 (18)	−0.0003 (15)	0.0163 (14)	0.0019 (15)
C5	0.0348 (19)	0.0283 (18)	0.0369 (16)	0.0054 (15)	0.0198 (14)	0.0042 (14)
C6	0.0331 (18)	0.0277 (17)	0.0263 (14)	0.0038 (15)	0.0130 (13)	0.0050 (12)
C7	0.0248 (17)	0.0256 (17)	0.0292 (15)	−0.0013 (14)	0.0094 (12)	0.0011 (12)
C8	0.0336 (19)	0.0287 (19)	0.0332 (16)	−0.0018 (15)	0.0132 (14)	0.0005 (13)
C9	0.0334 (19)	0.0242 (17)	0.0494 (18)	0.0038 (16)	0.0095 (15)	−0.0026 (16)
C10	0.0270 (18)	0.030 (2)	0.0446 (18)	0.0009 (15)	0.0011 (14)	0.0128 (15)
C11	0.0318 (19)	0.035 (2)	0.0344 (16)	0.0019 (16)	0.0061 (14)	0.0073 (14)
C12	0.0289 (18)	0.0275 (18)	0.0310 (15)	0.0014 (14)	0.0090 (13)	0.0025 (13)
C14	0.0313 (17)	0.0315 (18)	0.0215 (13)	0.0009 (15)	0.0079 (12)	0.0011 (12)
C15	0.0315 (18)	0.0294 (17)	0.0242 (14)	−0.0069 (14)	0.0113 (12)	−0.0064 (12)
C16	0.0303 (17)	0.0236 (16)	0.0300 (14)	0.0009 (15)	0.0144 (12)	−0.0033 (13)
C18	0.0249 (16)	0.0243 (16)	0.0281 (14)	0.0008 (14)	0.0131 (12)	0.0022 (12)
C19	0.0320 (19)	0.0294 (18)	0.0294 (15)	−0.0067 (14)	0.0121 (13)	−0.0010 (13)
C20	0.045 (2)	0.0235 (17)	0.0374 (17)	−0.0017 (16)	0.0145 (15)	0.0024 (14)
C21	0.038 (2)	0.032 (2)	0.0370 (17)	0.0044 (16)	0.0142 (15)	0.0112 (15)
C22	0.0351 (19)	0.041 (2)	0.0287 (15)	−0.0011 (16)	0.0078 (13)	0.0003 (13)
C23	0.0353 (19)	0.0239 (16)	0.0292 (15)	−0.0013 (14)	0.0094 (13)	−0.0004 (12)
C24	0.0267 (16)	0.0215 (15)	0.0277 (14)	0.0013 (13)	0.0137 (12)	0.0072 (12)
C25	0.0308 (18)	0.0331 (19)	0.0283 (15)	0.0019 (15)	0.0075 (13)	0.0037 (13)
C26	0.0234 (17)	0.040 (2)	0.0465 (19)	0.0029 (16)	0.0047 (14)	0.0070 (16)
C27	0.0310 (19)	0.045 (2)	0.051 (2)	0.0065 (17)	0.0230 (16)	0.0102 (17)
C28	0.036 (2)	0.056 (3)	0.0379 (18)	0.0045 (19)	0.0204 (15)	0.0033 (16)
C29	0.0315 (19)	0.048 (2)	0.0340 (16)	0.0007 (17)	0.0149 (14)	−0.0026 (15)
C30	0.0326 (19)	0.0322 (19)	0.0375 (17)	−0.0062 (16)	0.0164 (14)	−0.0004 (14)
C31	0.035 (2)	0.0329 (19)	0.0398 (17)	−0.0043 (17)	0.0143 (15)	−0.0019 (14)
O1	0.0560 (18)	0.0581 (19)	0.0394 (14)	−0.0176 (15)	0.0092 (12)	−0.0174 (13)
O2	0.0384 (16)	0.0501 (18)	0.0686 (18)	0.0134 (14)	0.0201 (13)	0.0018 (14)
O3	0.0305 (12)	0.0251 (11)	0.0260 (10)	−0.0031 (10)	0.0126 (9)	−0.0013 (8)
O4	0.0270 (11)	0.0290 (13)	0.0264 (10)	−0.0060 (9)	0.0141 (8)	−0.0047 (8)

### Geometric parameters (Å, °)

**Table:**

Fe1—C31	1.763 (4)	C14—C15	1.513 (5)
Fe1—C30	1.765 (3)	C14—H14A	0.9700
Fe1—P2	2.1414 (10)	C14—H14B	0.9700
Fe1—P1	2.1462 (10)	C15—C16	1.509 (4)
P1—O3	1.636 (2)	C15—H15A	0.9700
P1—C1	1.824 (3)	C15—H15B	0.9700
P1—C7	1.834 (3)	C16—O4	1.443 (4)
P2—O4	1.625 (2)	C16—H16A	0.9700
P2—C24	1.816 (3)	C16—H16B	0.9700
P2—C18	1.832 (3)	C18—C23	1.389 (4)
C1—C2	1.390 (4)	C18—C19	1.390 (5)
C1—C6	1.402 (4)	C19—C20	1.382 (5)
C2—C3	1.387 (5)	C19—H19	0.9300
C2—H2	0.9300	C20—C21	1.389 (5)
C3—C4	1.382 (5)	C20—H20	0.9300
C3—H3	0.9300	C21—C22	1.377 (5)
C4—C5	1.378 (5)	C21—H21	0.9300
C4—H4	0.9300	C22—C23	1.391 (5)
C5—C6	1.378 (5)	C22—H22	0.9300
C5—H5	0.9300	C23—H23	0.9300
C6—H6	0.9300	C24—C25	1.394 (4)
C7—C12	1.395 (4)	C24—C29	1.395 (4)
C7—C8	1.399 (5)	C25—C26	1.386 (5)
C8—C9	1.382 (5)	C25—H25	0.9300
C8—H8	0.9300	C26—C27	1.395 (5)
C9—C10	1.385 (5)	C26—H26	0.9300
C9—H9	0.9300	C27—C28	1.369 (5)
C10—C11	1.385 (5)	C27—H27	0.9300
C10—H10	0.9300	C28—C29	1.377 (5)
C11—C12	1.387 (5)	C28—H28	0.9300
C11—H11	0.9300	C29—H29	0.9300
C12—H12	0.9300	C30—O1	1.152 (4)
C14—O3	1.440 (4)	C31—O2	1.150 (4)
			
C31—Fe1—C30	101.01 (16)	C15—C14—H14A	109.9
C31—Fe1—P2	96.31 (12)	O3—C14—H14B	109.9
C30—Fe1—P2	115.62 (12)	C15—C14—H14B	109.9
C31—Fe1—P1	99.98 (12)	H14A—C14—H14B	108.3
C30—Fe1—P1	117.64 (11)	C16—C15—C14	112.6 (2)
P2—Fe1—P1	119.37 (4)	C16—C15—H15A	109.1
O3—P1—C1	96.34 (13)	C14—C15—H15A	109.1
O3—P1—C7	101.98 (13)	C16—C15—H15B	109.1
C1—P1—C7	99.68 (14)	C14—C15—H15B	109.1
O3—P1—Fe1	117.47 (9)	H15A—C15—H15B	107.8
C1—P1—Fe1	118.84 (10)	O4—C16—C15	108.8 (2)
C7—P1—Fe1	118.60 (11)	O4—C16—H16A	109.9
O4—P2—C24	96.22 (13)	C15—C16—H16A	109.9
O4—P2—C18	101.85 (13)	O4—C16—H16B	109.9
C24—P2—C18	102.69 (14)	C15—C16—H16B	109.9
O4—P2—Fe1	119.27 (9)	H16A—C16—H16B	108.3
C24—P2—Fe1	116.88 (11)	C23—C18—C19	118.8 (3)
C18—P2—Fe1	116.62 (11)	C23—C18—P2	121.6 (3)
C2—C1—C6	118.4 (3)	C19—C18—P2	119.6 (2)
C2—C1—P1	120.6 (2)	C20—C19—C18	120.9 (3)
C6—C1—P1	120.9 (2)	C20—C19—H19	119.6
C3—C2—C1	120.6 (3)	C18—C19—H19	119.6
C3—C2—H2	119.7	C19—C20—C21	119.9 (3)
C1—C2—H2	119.7	C19—C20—H20	120.0
C4—C3—C2	120.4 (3)	C21—C20—H20	120.0
C4—C3—H3	119.8	C22—C21—C20	119.6 (3)
C2—C3—H3	119.8	C22—C21—H21	120.2
C5—C4—C3	119.3 (3)	C20—C21—H21	120.2
C5—C4—H4	120.4	C21—C22—C23	120.5 (3)
C3—C4—H4	120.4	C21—C22—H22	119.7
C6—C5—C4	121.0 (3)	C23—C22—H22	119.7
C6—C5—H5	119.5	C18—C23—C22	120.2 (3)
C4—C5—H5	119.5	C18—C23—H23	119.9
C5—C6—C1	120.3 (3)	C22—C23—H23	119.9
C5—C6—H6	119.9	C25—C24—C29	118.3 (3)
C1—C6—H6	119.9	C25—C24—P2	122.0 (2)
C12—C7—C8	118.5 (3)	C29—C24—P2	119.8 (2)
C12—C7—P1	120.9 (3)	C26—C25—C24	120.7 (3)
C8—C7—P1	120.4 (2)	C26—C25—H25	119.6
C9—C8—C7	120.9 (3)	C24—C25—H25	119.6
C9—C8—H8	119.6	C25—C26—C27	119.9 (3)
C7—C8—H8	119.6	C25—C26—H26	120.1
C8—C9—C10	119.8 (3)	C27—C26—H26	120.1
C8—C9—H9	120.1	C28—C27—C26	119.5 (3)
C10—C9—H9	120.1	C28—C27—H27	120.3
C9—C10—C11	120.3 (3)	C26—C27—H27	120.3
C9—C10—H10	119.8	C27—C28—C29	120.9 (3)
C11—C10—H10	119.8	C27—C28—H28	119.5
C10—C11—C12	119.8 (3)	C29—C28—H28	119.5
C10—C11—H11	120.1	C28—C29—C24	120.7 (3)
C12—C11—H11	120.1	C28—C29—H29	119.6
C11—C12—C7	120.6 (3)	C24—C29—H29	119.6
C11—C12—H12	119.7	O1—C30—Fe1	177.8 (3)
C7—C12—H12	119.7	O2—C31—Fe1	178.6 (3)
O3—C14—C15	109.1 (3)	C14—O3—P1	120.4 (2)
O3—C14—H14A	109.9	C16—O4—P2	121.75 (19)
